# Impact of conduction disturbances on left ventricular mass regression and geometry change following transcatheter aortic valve replacement

**DOI:** 10.1038/s41598-021-96297-5

**Published:** 2021-08-18

**Authors:** Tsung-Yu Ko, Hsien-Li Kao, Ying-Ju Liu, Chih-Fan Yeh, Ching-Chang Huang, Ying-Hsien Chen, Chi-Sheng Hung, Chih-Yang Chan, Lung-Chun Lin, Yih-Sharng Chen, Mao-Shin Lin

**Affiliations:** 1 Department of Internal Medicine and Cardiovascular Center, National Taiwan University Hospital, Taipei, Taiwan; 2 Graduate Institute of Clinical Medicine, Medical College, National Taiwan University, Taipei, Taiwan; 3 Department of Internal Medicine, National Taiwan University Hospital, 7 Chung-Shan South Road, Taipei, Taiwan; 4 Department of Anesthesiology, National Taiwan University Hospital, Taipei, Taiwan; 5 Department of Surgery, National Taiwan University Hospital, Taipei, Taiwan

**Keywords:** Interventional cardiology, Cardiac device therapy

## Abstract

Our study aimed to compare the difference of LV mass regression and remodeling in regard of conduction disturbances (CD) following transcatheter aortic valve replacement (TAVR). A prospective analysis of 152 consecutive TAVR patients was performed. 53 patients (34.9%) had CD following TAVR, including 30 (19.7%) permanent pacemaker implantation and 23 (15.2%) new left bundle branch block. In 123 patients with 1-year follow-up, significant improvement of LV ejection fraction (LVEF) (baseline vs 12-month: 65.1 ± 13.2 vs 68.7 ± 9.1, P = 0.017) and reduced LV end-systolic volume (LVESV) (39.8 ± 25.8 vs 34.3 ± 17.1, P = 0.011) was found in non-CD group (N = 85), but not in CD group (N = 38). Both groups had significant decrease in LV mass index (baseline vs 12-month: 148.6 ± 36.9 vs. 136.4 ± 34.7 in CD group, p = 0.023; 153.0 ± 50.5 vs. 125.6 ± 35.1 in non-CD group, p < 0.0001). In 46 patients with 3-year follow-up, only non-CD patients (N = 28) had statistically significant decrease in LV mass index (Baseline vs 36-month: 180.8 ± 58.8 vs 129.8 ± 39.1, p = 0.0001). Our study showed the improvement of LV systolic function, reduced LVESV and LV mass regression at 1 year could be observed in patients without CD after TAVR. Sustained LV mass regression within 3-year was found only in patients without CD.

## Introduction

With the improvements in technology and the advent of minimalist approach, the rate of periprocedural complications during transcatheter aortic valve replacement (TAVR) has decreased over time^1–3^. Unfortunately, the incidence of conduction disturbances requiring permanent pacemaker (PM) implantation, and new-onset left bundle-branch block (LBBB) have not changed significantly, with reports even suggesting an increased risk after the introduction of newer generation transcatheter valves (THV)^4–7^. Both LBBB and PM implantation were known to be associated with left ventricular (LV) dyssynchrony and ventricular remodeling, and then result in impairment of LV function in long term follow-up^8–10^. It is mandatory to know the long-term effect of TAVR-induced conduction disturbance (CD) to LV function and geometry, because TAVR is set to expand to younger age and low surgical risk population.

New onset of CD has been associated with a decreased recovery of LV ejection fraction (LVEF) and a less favorable LV remodeling 6 to 12 months after TAVR, but few data exists regarding the impact to LV mass regression and remodeling^11–17^. The aim of this study was to compare the difference of LV function, remodeling and mass regression between the patients with and without CD following TAVR.

## Methods

### Patient population

170 consecutive patients (77 male, mean age 81.5 ± 6.9 years) with severe symptomatic aortic stenosis (AS), with extreme or high surgical risks, underwent TAVR in National Taiwan University Hospital from September 2010 to November 2017. Of them, a total of 18 patients were excluded for the following reasons: peri-procedural death (n = 2), previous PM implantation (n = 7), missing data in electrocardiogram and echocardiogram (n = 4), lost follow-up within 1 year (n = 3), THV explant 6 months after TAVR due to infected endocarditis (n = 1), and TAVR for failed surgical prosthesis (n = 1). A total of 152 patients were finally included.

### Ethical approval statement

All patients had sign informed consent at our clinic, and all the clinical information were collected according the protocol of Asian TAVR registry (NCT02308150) which was also proved by the local institutional review board of National Taiwan University Hospital. The evaluation and management of above patients were carried out in accordance with current guideline of valvular heart disease^18^.

### TAVR procedure

The TAVR was performed by a heart team composed of interventional cardiologists, cardiac surgeons, echocardiographer, and anesthesiologist. The pre-TAVR evaluation included transthoracic echocardiography and computed tomography (CT). The area and perimeter of the aortic valve annulus were analyzed through CT scan for selection of optimal valve size. The patients were implanted with CoreValve/Evolute R (Medtronic, Minneapolis, Minnesota) (n = 110, 72.3%), Sapien XT (Edwards Lifesciences, Irvine LLC, California) (n = 31, 20.4%), Lotus (Boston Scientific, Marlborough, Massachusetts) (n = 10, 6.6%) or Portico (Abbott Vascular, St. Paul, MN, USA) (n = 1, 0.7%) respectively. The depth of implantation was assessed by the angiography with angle vertical to the alignment of valve prosthesis, and was determined by the distance between the bottom of metallic frame and lowest annulus. The definitions of low implantation are: 6 mm below annular plane in CoreValve/Evolute R^19^, and 4 mm in Sapien XT, Lotus, or Portico^20^.

### Post-TAVR follow-up including electrocardiogram and echocardiography

Electrocardiographic records were obtained from all patients at baseline, immediately after the procedure, and daily until hospital discharge. The diagnosis of intraventricular conduction abnormalities was based on American Heart Association/American College of Cardiology Foundation/Heart Rhythm Society recommendations for the standardization and interpretation of the electrocardiogram^21^. New-onset LBBB was defined as any new LBBB occurring during the hospitalization period after the TAVR procedure that persisted at hospital discharge. PM implantation was indicated if third-degree or advanced second-degree atrioventricular block was found at any anatomic level that was not expected to resolve after the intervention and for sinus node dysfunction with documented symptomatic bradycardia, in agreement with the American Heart Association/ American College of Cardiology Foundation/Heart Rhythm Society guidelines for device-based therapy of cardiac rhythm abnormalities^19^. The indication of PM implantation in the presence of LBBB with PR prolongation (> 200 ms) not expected to normalize was at the discretion of the physician. The selection of a single-chamber or dual-chamber pacemaker was left to the implanter. The definition of new CD was either documentation of a new-onset LBBB or the new need of PM before discharge.

Transthoracic echocardiography examinations were performed at baseline, at 1 and 6-month, and annually during follow-up. Echocardiographic parameters included LV end-systolic dimension (LVESd) and volume (LVESV), LV end-diastolic dimension (LVEDd) and volume (LVEDV), septal wall thickness at end diastole (SWTd) and posterior wall thickness at end diastole (PWTd), LV ejection fraction (LVEF), aortic valve area (AVA), trans-aortic valve peak and mean pressure gradient were measured. The LVEDV and LVEF were measured by the biplane Simpson’s method from apical views. The AVA was estimated by the 2-dimensional Doppler method using the continuity equation. The LV mass was calculated by the formula as shown below:

LV mass = 0.8 × {1.04 [(LVEDd + PWTd + SWTd)^3 ^− (LVEDd)^3^]} + 0.6 g.

The LV mass index (LVMi) was calculated by LV mass indexed to body surface area^22^.

### Statistical analysis

Categorical data were presented as numbers (percentages) and compared using the Fisher’s exact tests or Pearson’s Chi-square test. Continuous variables were expressed as mean ± SD and were tested for normality using the Shapiro-Wilks test. The multivariate analysis were adjusted for baseline differences in the univariate analysis including variables with a value of *P* ≤ 0.20, such as gender, diabetes mellitus, previous myocardial infarction, LVEDd, valve type and low implantation, and using stepwise method. Comparisons between two groups were performed using independent Student’s t tests and pairwise data were compared by the paired t test if data were normally distributed. Pairwise comparisons of non-normally distributed data were tested with Wilcoxon’s signed-rank tests and analyzing of independent variables was realized by Mann–Whitney U test. Generalized estimated equations for repeated measurement were performed to evaluate temporal changes in LVMi before and after TAVR. The results were considered significant at values of *P* < 0.05. Data analyzed with STATA version 14.2.

## Results

In 152 patients, 4 patients (2.6%) had baseline LBBB and 16 patients (10.5%) had baseline right bundle branch block. After TAVR, new onset of CD occurred in 53 patients (34.9%) before discharge, including 30 (19.7%) new PM and 23 (15.2%) new LBBB. In 4 patients having prior LBBB, 3 had new PM implantation after TAVR (classified as new CD group) and 1 kept LBBB during follow-up (classified as non-CD group). All of PMs implanted were dual-chamber PM. 25 patients received new PM due to complete atrioventricular block, and another 5 patients due to sinus node dysfunction. Baseline characteristic and procedural variables of the study population according to new onset of CD after TAVR are shown in Table 1. Factors associated with new CD after TAVR in univariate analysis were male gender (CD group vs non-CD group: 69.8% vs. 49.5%, p = 0.017), CoreValve/Evolute-R (44.7% vs. 24.0%, p = 0.01), and low implantation (43.4% vs 18.2%, P = 0.003). There was a trend toward older age in CD group (82.5 ± 7.3 vs 80.5 ± 6.9, P = 0.091), but not statistically significant. In the multivariate analysis (Table 2), male gender (hazard ratio: 0.36; 95% confidence interval: 0.15 to 0.87; P = 0.031) and low implantation (hazard ratio: 1.68; 95% CI 1.04 to 2.70; P = 0.032) were independent predictors of post-TAVR new CD.

**Table 1:** Baseline characteristic and procedural variables according to CD after TAVR.

	CD (N = 53)	Non-CD (N = 99)	P value
Age (years)	82.5 ± 7.3	80.5 ± 6.9	0.091
Male (%)	37(69.8)	49(49.5)	0.017
STS score	7.5 ± 5.8	7.3 ± 4.9	0.813
Body mass index (kg/m^2^)	24.1 ± 3.8	23.6 ± 5.7	0.551
NYHA Fc III and IV (%)	48(80.6)	82(82.8)	0.478
Diabetes mellitus (%)	24(45.2)	29(29.2)	0.146
Hypertension (%)	34(64.2)	70(70.7)	0.407
Hyperlipidemia (%)	14(26.4)	35(35.5)	0.281
Previous myocardial infarction (%)	5(9.4)	4(4.1)	0.179
Previous CABG (%)	2(1.9)	4(4.1)	0.657
LVEF(%)	66.6 ± 12.0	65.3 ± 12.9	0.552
LVEDd (mm)	45.9 ± 8.1	48.0 ± 6.8	0.086
Ao max PG (mmHg)	78.2 ± 23.5	76.9 ± 28.5	0.764
Ao mean PG (mmHg)	45.4 ± 14.8	44.7 ± 17.7	0.811
Aortic valve area (cm^2^)	0.71 ± 0.19	0.71 ± 0.17	0.993
LVMi (g/m^2^)	152.7 ± 36.4	151.7 ± 48.9	0.894
Valve type			0.002
CoreValve + Evolut R (%)	46(86.7)	64(64.6)	
Lotus (%)	4(7.6)	6(6.1)	
Sapien XT (%)	3(5.7)	28(28.3)	
Portico (%)	0	1(1.0)	
Valve size (mm)			0.333
31 (%)	4(7.6)	2(2.1)	
29/27(%)	16(30.2)	36(36.4)	
26/25(%)	27(50.9)	48(48.4)	
23 (%)	6(11.3)	13(13.1)	
Annulus perimeter (mm)	71.5 ± 7.6	72.8 ± 7.4	0.375
Oversizing (%)*	19.1 ± 7.0	18.0 ± 7.3	0.519
Post-dilatation (%)	11(20.7)	21(21.2)	0.947
Low implantation (%)	23(43.4)	18(18.2)	0.003

Mean ± SD are shown.

*CD* conduction disturbance, *CABG* coronary artery bypass graft surgery, *STS* society of thoracic surgeon, *NYHA* New York heart association, *LVEF* left ventri-cular ejection fraction, *LVEDd* left ventricular end-diastolic dimension, *IVS* inter-ventricular septum, *PW* posterior wall, *Ao max PG* aortic valve maximal pressure gradient, *Ao mean PG* aortic valve mean pressure gradient, *LVMi* left ventricular mass index.

*Oversizing (%) = [(Valve perimeter /Annulus perimeter)  − 1] × 100 for self-expanding valve and mechanical-expanding valve; [(Valve area/Annulus area)-1] × 100 for balloon-expandable valve.

**Table 2:** Multivariate analysis for predictors of post-TAVR conduction disturbance.

	Hazard ratio	95% confidence interval	P value
Male gender	0.36	(0.15–0.87)	0.031
Low implantation	1.68	(1.04–2.70)	0.032
Self-expanding valve	2.50	(0.86–7.30)	0.093
Diabetes mellitus	1.79	(0.943–3.39)	0.075

In 99 patients without TAVR-related CD, 4 patients (4.0%) developed new LBBB and 3 patients (3.0%) developed new PM implantation within 1 year follow-up. In 23 patients with TAVR-related LBBB, 4 patients (17.4%) developed new PM implantation, and 4 patients (17.4%) had resolution of LBBB within 1 year follow-up. During 1- to 3-year follow-up, neither new conduction disorders nor resolution of LBBB were observed.

### CD vs. non-CD during 1-year follow-up

**Figure 1:**
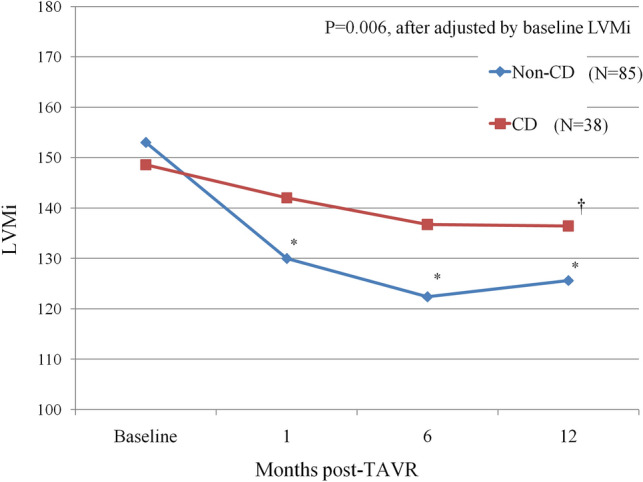
Temporal changes of LVMi before and after TAVR according to new onset of conduction disturbance in patients completing 1-year echocardiographic follow-up. *CD* conduction disturbance, *LVMi* left ventricular mass index, *TAVR* transcatheter aortic valve replacement. *P < 0.0001 as compared to baseline data. ^†^P = 0.023 as compared to baseline data.

Among 152 patients, 15 patients died [8 (15.1%) in CD group and 7 (7.1%) in non-CD group] and 14 patients did not receive 12-month echocardiographic evaluation [7 (13.2%) in CD group and 7 (7.1%) in non-CD group]. A total of 123 patients completed 12 month echocardiographic evaluation, with 38 patients (30.9%) having new CD after TAVR and 85 (69.1%) without. At baseline, there were no statistical differences in any echocardiographic parameters between patients with and without new CD after TAVR (Table 3). At 1-year follow-up, CD group had smaller AVA (cm^2^) (CD vs non-CD group: 1.59 ± 0.20 vs 1.71 ± 0.23, P = 0.011) as compared to non-CD group. LVMi (g/m^2^) (CD group: baseline vs 1-year: 148.6 ± 36.9 vs 136.4 ± 34.7, P = 0.023; Non-CD group: 153.0 ± 50.5 vs 125.6 ± 35.1, P < 0.0001), SWTd (mm) (CD group: baseline vs 12-month: 13.2 ± 2.1 vs 12.3 ± 2.1, P = 0.018; Non-CD group: 12.9 ± 2.4 vs 11.4 ± 2.0, P < 0.0001) and PWTd (mm) (CD group: baseline vs 12-month: 12.7 ± 2.0 vs 11.8 ± 1.8, P = 0.006; Non-CD group: 12.3 ± 2.2 vs 10.9 ± 1.9, P < 0.0001) all reduced significantly in both groups. In respect of LVMi changes from baseline to 1 year, a borderline difference between CD and non-CD group was observed. (△LVMi: CD group vs non-CD group: − 12.2 ± 32.7 vs − 27.7 ± 47.7 g/m^2^, P = 0.067). Figure 1 shows the temporal changes of LVMi before and after TAVR according to TAVR-related CD. In non-CD group, sustained reduction of LVMi from baseline to 1, 6 and 12 month (all P values < 0.0001 as comparing to baseline) could be observed. But in CD group, significant reduction of LVMi could only be observed from baseline to 12 months. After adjusted by baseline LVMi, there is significant difference between CD and non-CD group in 1-year follow-up with the P value = 0.006. Otherwise, significant improvement of LVEF (%) (Baseline vs 1-year: 65.1 ± 13.2 vs 68.7 ± 9.1, P = 0.017), reduction of LVESd (mm) (30.7 ± 8.0 vs 29.1 ± 5.6, P = 0.021) and LVESV (mm) (39.8 ± 25.8 vs 34.3 ± 17.1, P = 0.011) were observed only in non-CD, but not in CD group.

**Table 3:** Baseline and 1-year postprocedural echocardiographic parameters according to CD in patients complete 1-years follow-up.

	CD (N = 38)		P value	Non-CD (N = 85)		P value
	Baseline	1 Year follow-up		Baseline	1 Year follow-up	
LVEF (%)	67.9 ± 11.7	67.5 ± 10.4	0.813	65.1 ± 13.2	68.7 ± 9.1	0.007
Ao mean PG (mmHg)	47.4 ± 14.4	9.3 ± 5.2	< 0.0001	43.5 ± 18.0	9.0 ± 5.7	< 0.0001
Aortic valve area (cm^2^)	0.72 ± 0.17	1.59 ± 0.20	< 0.0001	0.72 ± 0.18	1.71 ± 0.23*	< 0.0001
LVESd (mm)	28.3 ± 8.1	29.1 ± 6.8	0.524	30.7 ± 8.0	29.1 ± 5.6	0.021
LVEDd (mm)	45.8 ± 7.8	46.8 ± 7.3	0.355	48.2 ± 6.8	47.7 ± 6.3	0.456
SWTd (mm)	13.2 ± 2.1	12.3 ± 2.1	0.018	12.9 ± 2.4	11.4 ± 2.0	< 0.0001
PWTd (mm)	12.7 ± 2.0	11.8 ± 1.8	0.006	12.3 ± 2.2	10.9 ± 1.9	< 0.0001
LVMi (g/m^2^)	148.6 ± 36.9	136.4 ± 34.7	0.023	153.0 ± 50.5	125.6 ± 35.1	< 0.0001
LVEDV (ml)	100.8 ± 38.9	101.6 ± 37.6	0.886	111.3 ± 35.9	108.9 ± 32.6	0.463
LVESV (ml)	36.5 ± 26.1	35.5 ± 20.3	0.761	39.8 ± 25.8	34.3 ± 17.1	0.011

Mean ± SD are shown.

*CD* conduction disturbance, *LVEF* left ventricular ejection fraction, *LVESd* left ventricular end-systolic dimension, *LVEDd* left ventricular end-diastolic dimension, *PWTd* posterior wall thickness at end diastole, *SWTd* septal wall thickness at end diastole, *LVMi* left ventricular mass index, *LVEDV* left ventricular end-diastolic volume, *LVESV* left ventricular end-systolic volume.

*P < 0.05 as compared to 1 year data in CD group.

In 33 patients receiving new PM within 1 year post-TAVR, 16 patients received regular PM follow-up at our hospital. There were 12 patients (12/16, 75%) required more than 99% of ventricular pacing. 2 patients had less than 1% ventricular pacing, and another 2 patients had 43%, 22% ventricular pacing respectively.

### CD vs. non-CD during 3-year follow-up

**Figure 2:**
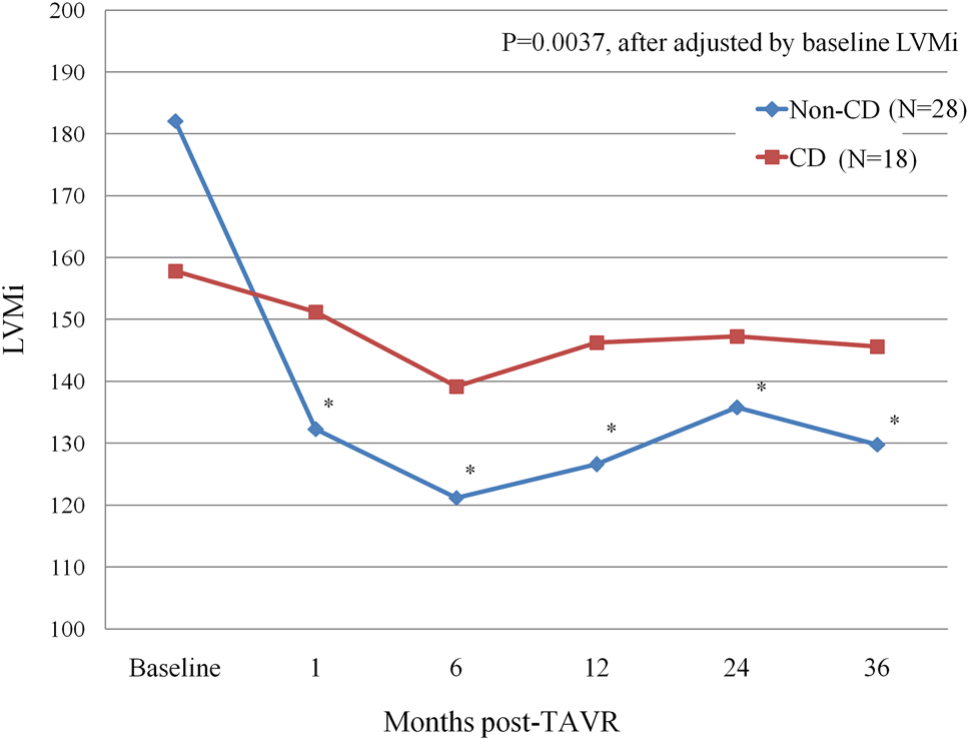
Temporal changes of LVMi before and after TAVR according to new onset of conduction disturbance in patients completing 3-year echocardiographic follow-up. *CD* conduction disturbance, *LVMi* left ventricular mass index, *TAVR* transcatheter aortic valve replacement. *P < 0.001 as compared to baseline data.

77 patients have received TAVR procedure for 3 years [32 (41.6%) in CD group and 45 (58.4%) in non-CD group]; among these patients, 24 patients died [10 (10/32, 31.3%) in CD group and 14 (14/45, 31.1%) in non-CD group] and 7 patients did not receive echocardiographic evaluation [4 (4/32, 12.5%) in CD group and 3 (3/45, 6.7%) in non-CD group]. A total 46 patients completed 3 year echocardiographic evaluation, 18 patients (39.1%) with new CD after TAVR and 28 (60.9%) without. Baseline LVEF (%) before TAVR was better in CD group (CD vs Non-CD: 70.7 ± 10.5 vs 62.5 ± 14.9, P = 0.043),with significantly smaller LVESd (mm) (27.4 ± 8.0 vs 33.5 ± 8.9, P = 0.026) and LVESV (ml) (32.2 ± 22.0 vs 50.8 ± 31.5, P = 0.024) (Table 4). At 3-year follow-up, PWTd (mm) (CD group: baseline vs 3-year: 13.5 ± 1.7 vs 11.7 ± 2.1, P = 0.0004; Non-CD group: 12.9 ± 2.1 vs 11.3 ± 1.6, P = 0.004) reduced significantly in both groups, but significant LVMi (g/m^2^) reduction (Baseline vs 3-year: 180.8 ± 58.8 vs 129.8 ± 39.1, P = 0.0001), SWTd (mm) reduction (13.9 ± 2.1 vs 11.3 ± 1.5, P < 0.0001) and LVEDd (mm) reduction (50.8 ± 7.2 vs 47.8 ± 7.3, P = 0.041) were observed only in non-CD group, with also a trend toward reduced LVEDV (ml) (125.5 ± 39.1 vs 110.5 ± 39.5, P = 0.082) and decreased LVESV (ml) (50.8 ± 31.5 vs 41.1 ± 29.7, P = 0.191). Figure 2 shows the temporal changes of LVMi before and after TAVR according to TAVR-related CD. In non-CD group, sustained reduction of LVMi from baseline to 1, 6, 12, 24 and 36 months (P < 0.001 as comparing to baseline) could be observed, whereas LVMi remained stable over time in CD group. After adjusted by baseline LVMi, there is significant difference between CD and non-CD group in 3-year follow-up with the P value = 0.0037.

**Table 4:** Baseline and 3-year postprocedural echocardiographic parameters according to CD in patients complete 3-years follow-up.

	CD (N = 18)			Non-CD (N = 28)		P value
	Baseline	3 Year follow-up		Baseline	3 Year follow-up	
LVEF (%)	70.7 ± 10.5	68.2 ± 8.3	0.446	62.5 ± 14.9*	62.3 ± 16.9	0.770
Ao mean PG (mmHg)	51.2 ± 14.3	8.8 ± 4.2	< 0.0001	45.8 ± 15.6	8.0 ± 3.1	< 0.0001
Aortic valve area (cm^2^)	0.67 ± 0.18	1.64 ± 0.21	< 0.0001	0.66 ± 0.18	1.66 ± 0.17	< 0.0001
LVESd (mm)	27.4 ± 8.0	29.4 ± 5.1	0.156	33.5 ± 8.9*	31.4 ± 9.3	0.353
LVEDd (mm)	45.4 ± 8.3	47.6 ± 5.2	0.191	50.8 ± 7.2	47.8 ± 7.3	0.041
SWTd (mm)	13.9 ± 1.8	12.7 ± 2.1	0.071	13.9 ± 2.1	11.3 ± 1.5	< 0.0001
PWTd (mm)	13.5 ± 1.7	11.7 ± 2.1	0.0004	12.9 ± 2.1	11.3 ± 1.6	0.004
LVMi(g/m^2^)	157.8 ± 39.6	145.6 ± 40.1	0.191	180.8 ± 58.8	129.8 ± 39.1	0.0001
LVEDV (ml)	99.3 ± 40.8	107.0 ± 26.8	0.231	125.5 ± 39.1	110.5 ± 39.5	0.082
LVESV (ml)	32.2 ± 22.0	34.5 ± 13.6	0.277	50.8 ± 31.5*	41.1 ± 29.7	0.191

Mean ± SD are shown.

*CD* conduction disturbance, *LVEF* left ventricular ejection fraction, *LVESd* left ventricular end-systolic dimension, *LVEDd* left ventricular end-diastolic dimension, *PWTd* posterior wall thickness at end diastole, *SWTd* septal wall thickness at end diastole, *LVMi* left ventricular mass index, *LVEDV* left ventricular end-diastolic volume, *LVESV* left ventricular end-systolic volume.

*P < 0.05 as compared to baseline data in CD group.

## Discussion

It was already known that new CD after TAVR had negative effect on LV function recovery^11–15^, and also had a trend toward a lower rate of LV reverse remodeling at 1-year follow-up^13^. Hoffmann et al. also reported that 1-year change in LVESV was significantly different between the patients with and without new CD^14^. Dimitriadis et al.^15^ evaluated the impact of TAVR-related CD on myocardial performance with longer follow-up period (mean: 29.1 ± 16.9 months). They found that the changes both in LVESd and LVEDd were significantly different between these two groups. However, the impact of new CD on LV mass regression and remodeling, were less well described. The main findings of this study are: (1) clinical risk factors of new onset CD after TAVR are male sex and low THV implantation. (2) At 1-year, significant LV mass regression could be observed both in CD and non-CD group, but more pronounced in non-CD group. LVEF improvement as well as LVESd and LVESV reduction could only be found in the non-CD group. (3) At 3-year, sustained LV mass regression and a trend toward reduced LVEDV and LVESV could only be observed in the non-CD group. The results not only remind the operators to avoid low implantation during procedure, but also urge the device manufactures to make every effort to prevent new CD, especially as TAVR is now being extended to a younger and lower risk population.

Several clinical and experimental studies have established the potential adverse effects of long term LBBB and right ventricular (RV) pacing on LV function. Early activation of RV may lead to a decrease in cardiac output as well as intraventricular and interventricular dyssynchrony, thus resulting in LV systolic dysfunction and remodeling^8,9,22,23^. In addition, the asynchronous ventricular activation leads to redistribution of circumferential shortening and myocardial blood flow, and then results in myocardial hypoperfusion in the absence of flow-limiting coronary artery disease^24–26^. The detrimental effects on LV geometry and function have been considered as one of possible mechanisms to explain why poorer functional status was observed in the population with post-TAVR LBBB, which has been illustrated in several studies^27^. The hypothesis is also congruent with observations that in chronic right ventricular pacing, heart failure hospitalization occurs more frequently in patients with depressed systolic function than in patients with normal systolic function^28^.

Dobson et al.^29^ using cardiac magnetic resonance, evaluated 24 patients with new LBBB following TAVR, matched with 24 patients with a narrow post-procedure QRS. Similar to our findings, significant improvement of LVEDV as well as reduced indexed LVESV at 6-months were seen only in narrow QRS group but not in the post-TAVR LBBB group. The authors concluded that TAVR-induced LBBB is associated with less favorable cardiac reverse remodeling at medium term follow up. However, these results were limited by its small case numbers and short follow-up period to provide longer term evidence of the adverse impact of new CD on cardiac reverse remodeling.

The difference of LV mass regression in long term follow-up between the patients with and without CD could be considered as a consequence of different geometric change after TAVR. In the first year after TAVR, the LV mass regression mostly resulted from the decrease of LV wall thickness in response to increased AVA and LV unloading, which could be demonstrated by our findings and previous study^30,31^. Interestingly though, the LV mass regression was more pronounced in non-CD group (CD vs non-CD group: 8% vs 18%) despite similar post-TAVR AVA in both groups. Less LV mass regression in CD group may result from the slight increase in LVEDd and LVEDV at 1 year, in contrast to the slight reduction of LVEDd and LVEDV in non-CD group. The effect of LV dimension and volume change to mass regression became more pronounced at 3-year follow-up. In non-CD group, sustained LV mass regression was observed along with sustained LVEDd and LVEDV reduction. The stationary (or slightly increasing) LVEDd and LVEDV in CD group, on the contrary, offset the wall thickness reduction and resulted in less LV mass regression. Our results may also provide explanation to the more pronounced LV mass regression observed in surgical aortic valve replacement compared with TAVR in prior randomized study^30^, as the incidence of CD was higher in TAVR group using self-expanding prosthesis.

## Limitations

The main limitation of the present study was its small sample size, especially case numbers completing 3-year follow-up. Selection bias might exist, and it is mandatory to prove the hypothesis in a larger population. Second, the clinical significance of reverse LV remodeling following TAVR remains to be established. Third, the pacing percentage and the rate of pacemaker dependence among the patients receiving PM were not fully investigated. According to previous literature, overall pacemaker dependence after TAVR varied from 27 to 68%^32^, and of intrinsic atrioventricular conduction increased from 25.9% at 7 days to 59.3% at 30 days^19^. Future larger study with longer term follow-up on the clinical outcome and detailed analysis of the PM recordings is mandatory. Furthermore, the patients who died within the interval of 1 year to 3 years follow-up might have experienced less LV mass regression and poorer LVEF improvement. Thus, the potential of a competitive risk bias could not be excluded.

## Conclusions

We concluded that in patients with severe AS receiving TAVR, the improvement of LV systolic function associated with decrease of LVESV at 1 year could be observed in non-CD group. Significant LV mass regression could be found in both groups at 1 year, but more pronounced in non-CD group. In limited patients completing 3-year echocardiographic follow-up, sustained LV mass regression and a trend toward reduced LVEDV and LVESV were observed in patients without post-TAVR CD.
